# Human Umbilical Cord Blood-Derived CD34^+^ Cells Reverse Osteoporosis in NOD/SCID Mice by Altering Osteoblastic and Osteoclastic Activities

**DOI:** 10.1371/journal.pone.0039365

**Published:** 2012-06-18

**Authors:** Reeva Aggarwal, Jingwei Lu, Suman Kanji, Matthew Joseph, Manjusri Das, Garrett J. Noble, Brooke K. McMichael, Sudha Agarwal, Richard T. Hart, Zongyang Sun, Beth S. Lee, Thomas J. Rosol, Rebecca Jackson, Hai-Quan Mao, Vincent J. Pompili, Hiranmoy Das

**Affiliations:** 1 Cardiovascular Stem Cell Research Laboratory, Davis Heart and Lung Research Institute, The Ohio State University Medical Center, Columbus, Ohio, United States of America; 2 Department of Biomedical Engineering, College of Engineering, The Ohio State University, Columbus, Ohio, United States of America; 3 Department of Physiology and Cell Biology, College of Medicine, The Ohio State University, Columbus, Ohio, United States of America; 4 Division of Oral Biology, Department of Orthopedics, College of Dentistry, The Ohio State University, Columbus, Ohio, United States of America; 5 Department of Veterinary Clinical Sciences, College of Veterinary Medicine, The Ohio State University, Columbus, Ohio, United States of America; 6 Division of Endocrinology, Diabetes and Metabolism, College of Medicine, The Ohio State University, Columbus, Ohio, United States of America; 7 Department of Materials Science and Engineering, John's Hopkins University, Baltimore, Maryland, United States of America; French Blood Institute, France

## Abstract

**Background:**

Osteoporosis is a bone disorder associated with loss of bone mineral density and micro architecture. A balance of osteoblasts and osteoclasts activities maintains bone homeostasis. Increased bone loss due to increased osteoclast and decreased osteoblast activities is considered as an underlying cause of osteoporosis.

**Methods and Findings:**

The cures for osteoporosis are limited, consequently the potential of CD34+ cell therapies is currently being considered. We developed a nanofiber-based expansion technology to obtain adequate numbers of CD34^+^ cells isolated from human umbilical cord blood, for therapeutic applications. Herein, we show that CD34^+^ cells could be differentiated into osteoblastic lineage, *in vitro*. Systemically delivered CD34^+^ cells home to the bone marrow and significantly improve bone deposition, bone mineral density and bone micro-architecture in osteoporotic mice. The elevated levels of osteocalcin, IL-10, GM-CSF, and decreased levels of MCP-1 in serum parallel the improvements in bone micro-architecture. Furthermore, CD34^+^ cells improved osteoblast activity and concurrently impaired osteoclast differentiation, maturation and functionality.

**Conclusions:**

These findings demonstrate a novel approach utilizing nanofiber-expanded CD34^+^ cells as a therapeutic application for the treatment of osteoporosis.

## Introduction

Osteoporosis is a systemic bone disorder, affecting more than 200 million people worldwide [1]. Bone is a dynamic organ that undergoes constant remodeling via cycles of bone formation and resorption, by osteoblasts and osteoclasts [2]. Imbalance of osteoclastic and/or osteoblastic activities generally results in low bone mineral density (BMD), loss of bone mass and mechanical strength, leading to increased risk of fractures, typical of osteoporosis [3]. Impaired osteoblastic differentiation of bone marrow progenitor cells may also play a significant role in developing osteoporosis. Age, endocrine malfunction or deficiency, nutrition, or lack of physical activity, all can imbalance the osteoblasts and osteoclasts activities, affecting both trabecular and cortical bone at molecular, cellular and structural levels [4], [5]. It has been shown that reduction in trabecular bone in osteoporosis is associated with increased adiposity in bone marrow, which could be due to transcriptional switch in favor of adipogenesis instead of osteoblastogenesis of bone marrow precursor cells [6], [7].

The mesenchymal progenitor cells in the bone marrow give rise to osteoblasts under the influence of multiple osteogenic signals specific for their proliferation and differentiation [8]. Osteoblastic differentiation is initiated by binding of bone morphogenetic proteins (BMPs) to their receptors that activate transcription factors, Runx2 and Osterix, and subsequent expression of downstream osteoblast specific genes such as alkaline phosphatase, collagen type 1, osteonectin, osteocalcin and bone sialoprotein [9], [10], [11]. BMPs upregulate osteoblastic genes *via* activation of Smad1/5/8 signaling molecules and regulate mineralization of osteoblastic cells via Wnt in an autocrine signaling loop [12]. Runx2 is a potent inhibitor of adipogenesis, and is required for the differentiation of adipocytes to osteogenic lineage [13]. Additionally, balance of osteoprotegrin (OPG): receptor activator of nuclear factor kappa-B ligand (RANKL) ratio, osteocalcin and cytokines such as interleukin (IL)-1 IL-4, IL-6, monocyte chemotactic protein (MCP)-1 and granulocyte macrophage colony stimulating factor (GM-CSF) have been shown to regulate the activities of osteoblastic and osteoclastic cells [14], [15].

Although, associated with side effects, anti-resorptive and anabolic therapies are currently available for osteoporosis [3], [16]. Furthermore, these therapies have temporary effects, and the decrease in fracture incidences in long-term is debatable [17], [18]. Recently, much effort has been expended to understand the therapeutic effectiveness of CD34+ cells in various degenerative diseases. However, the major hurdles are the unavailability of sufficient number of biologically functional CD34+ cells and maintaining their regenerative potential for therapeutic applications.

We previously reported that human CD133^+^/CD34^+^ cells could be expanded *in vitro* up to 250-fold in a serum-free medium on aminated poly-ether sulfone (PES) nanofiber coated plates within 10 days, while preserving stem cell phenotype and biological functionality [19]. These cells are considered biologically superior as they exhibit better engraftment capabilities, express homing markers (CXCR4 and LFA-1) towards bone marrow and maintain their multipotency. This allows them to differentiate into multiple lineages such as endothelial, and hematopoietic lineages. Here we show that nanofiber-expanded CD34^+^ cells could be differentiated towards osteoblastic lineage, *in vitro*. Furthermore, CD34^+^ cell transplantation into an osteoporotic NOD/SCID murine model augments bone formation rate, bone mineral density and improves bone micro-architecture. These improvements correlate with the elevated serum levels of osteocalcin, interleukin (IL)-10 and granulocyte-macrophage colony stimulating factor (GM-CSF), and decreased level of monocyte chemotactic protein-1 (MCP-1). CD34^+^ cell transplantation not only improved osteoblast functionality but also concurrently impaired differentiation and maturation of osteoclasts, thereby reducing osteoclast activity in osteoporotic mice. The findings demonstrate a novel potential of nanofiber-expanded CD34^+^ cells in reverting osteoporosis.

## Results

### Differentiation of nanofiber-expanded CD34^+^ cells towards osteoblastic lineage

**Figure 1:**
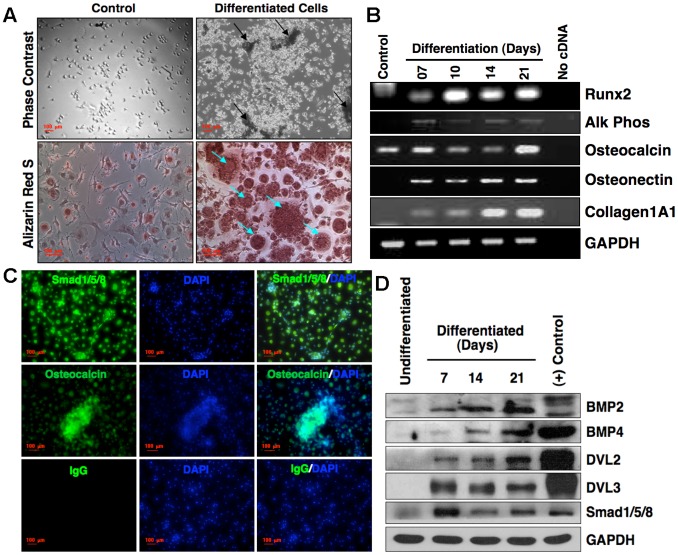
Osteoblastic differentiation of nanofiber-expanded CD34^+^ **cells. (A).** Morphology of the cells was visualized under phase contrast microscope at day 1 (control, upper panel) after seeding of nanofiber-expanded CD34^+^ cells (10 days expansion of CD133^+^/CD34^+^ cells on nanofiber) and at day 14 after differentiation of cells with osteoblast specific stimulants (b-glycerophosphate and ascorbic acid) in DMEM complete medium cultured on a 24-well tissue culture plate. Arrowheads indicate the clusters that formed after differentiation. Alizarin Red S staining (lower panel) was performed to the cells cultured for 21 days either with osteoblast specific differentiation medium (differentiated cells) or DMEM complete medium only (control). Red stains in the control image indicate the intracellular calcium in random differentiated cells and arrowheads indicate higher level of mineral depositions in differentiated cells. The experiment was repeated at least three times and representative images are shown. (**B**). RNA was isolated from differentiated cells during the course of osteoblastic differentiation at various time points as stated, and one microgram of RNA was used to make cDNA. One micro liter of cDNA was used to perform semi-quantitative RT-PCR analysis for Runx2, alkaline (Alk) phosphate, osteocalcin, osteonectin, collagen Type 1A1 and GAPDH as a loading control. Nanofiber expanded cells (10 days) were used as a control. (**C**). Detection of osteoblast specific proteins in differentiated cells**.** Immunocytochemical staining was performed with the 21 days-differentiated cells using either Sma and Mad related proteins (Smad 1/5/8) or osteocalcin specific antibodies, and IgG isotype as control. Green fluorescence indicates positive staining and blue fluorescence indicate DAPI (nuclear) staining. **(D)**. Protein levels were evaluated for various signaling molecules of BMP, Wnt and Smad pathways during the course of osteoblastic differentiation of nanofiber-expanded CD34+ cells. Undifferentiated nanofiber-expanded cells and differentiated MC3T3 cell line were used as controls. Representative of three sets of experiments is shown here.

Our previous studies showed that nanofiber expanded human umbilical cord blood (hUCB) CD34^+^ cells retain multipotency as evident by their ability to differentiate into endothelial or smooth muscle cells [19], [20]. Here we sought, whether these cells could also be differentiated towards osteoblastic lineage *in vitro*. To test that, CD133^+^ cells were isolated from hUCB and expanded on nanofiber coated plates in serum-free expansion medium with supplements for 10 days, *in vitro* [19]. The cell phenotype was confirmed by the expression of CD34, CD45, CXCR4, LFA-1, MHC-I & II, CD14, CD11a and absence of CD69, CD117, CD105 using flowcytometric analyses (data not shown). CD34+ cells were then induced to osteoblastic differentiation in the presence of ascorbic acid and b-glycerophosphate, *in vitro*. Remarkable cellular changes in shape and size were observed within 7 days and mineralized nodules were apparent at 21 days in more than 95% of the cells (Figure 1A, upper right panel). The mineral deposition by induced CD34^+^ cells was confirmed by the presence of calcium in Alizarin red stained cells. CD34^+^ cells cultured in tissue culture media (TCM) lacking ascorbic acid and b-glycerophosphate, also showed few randomly scattered cells with faint intracellular alizarin red staining (Figure 1A, lower left panel). However, the intensity of stain was markedly higher in cells induced to differentiate into osteoblastic lineages (Figure 1A, lower right panel).

### Molecular evidences for osteoblastic differentiation of CD34^+^ cells

To further investigate the differentiation of CD34^+^ cells into osteoblastic lineage, the expression of osteoblast specific genes and proteins were analyzed. Semi quantitative RT-PCR analysis revealed that differentiated nanofiber-expanded cells upregulated expression of transcription factor Runx2, and bone associated proteins such as alkaline phosphatase, osteocalcin, osteonectin and collagen type 1A1 at various time points during the course of differentiation (Figure 1B). The expression of osteocalcin in freshly isolated CD133^+^ cells was consistent with earlier reports [21]. Fold increase for the gene expressions at mRNA level compared to undifferentiated control was analyzed, as follows: Runx2; Day 7, 0.84±0.14; Day 21, 1.18±0.1; Alk Phos; Day 7, 0.9±0.05; Day 21, 1.7±0.13; Osteocalcin; Day 7, 1.02±0.05; Day 21, 1.56±0.1; Osteonectin; Day 7, 1.16±0.04; Day 21, 1.51±0.48; Collagen 1A1; Day 7, 0.61±0.13; Day 21, 1.79±0.27. Additionally, immunocytochemical analysis revealed that CD34^+^ cells stained positive with Smad1/5/8 and osteocalcin upto 21 days of differentiation (Figure 1C). Western blots showed an increase in protein levels of BMP2, BMP4, dishevelled protein (DVL) 2, DVL3 and Smad1/5/8 in differentiated cells at all time points during the course of differentiation. GAPDH was used as loading control and MC3T3 cells differentiated into osteoblasts were used as a positive control (Figure 1D). Fold increase of the protein level compared to undifferentiated control was analyzed, as follows BMP2; Day 7, 0.78±0.24; Day 21, 1.2±0.01; BMP4; Day 7, 0.45±0.2; Day 21, 1.7±0.61; Smad 1/5/8; Day 7, 1.46±0.04; Day 21, 1.01±0.12; DVL2; Day 7, 0.72±0.09; Day 21, 1.1±0.12; DVL3; Day 7, 1.24±0.04; Day 21, 1.0±0.12. Collectively, these data confirm osteoblastic differentiation of nanofiber expanded hUCB CD34^+^ cells.

### Nanofiber-expanded CD34^+^ cells induce bone formation in a murine model of osteoporosis

**Figure 2:**
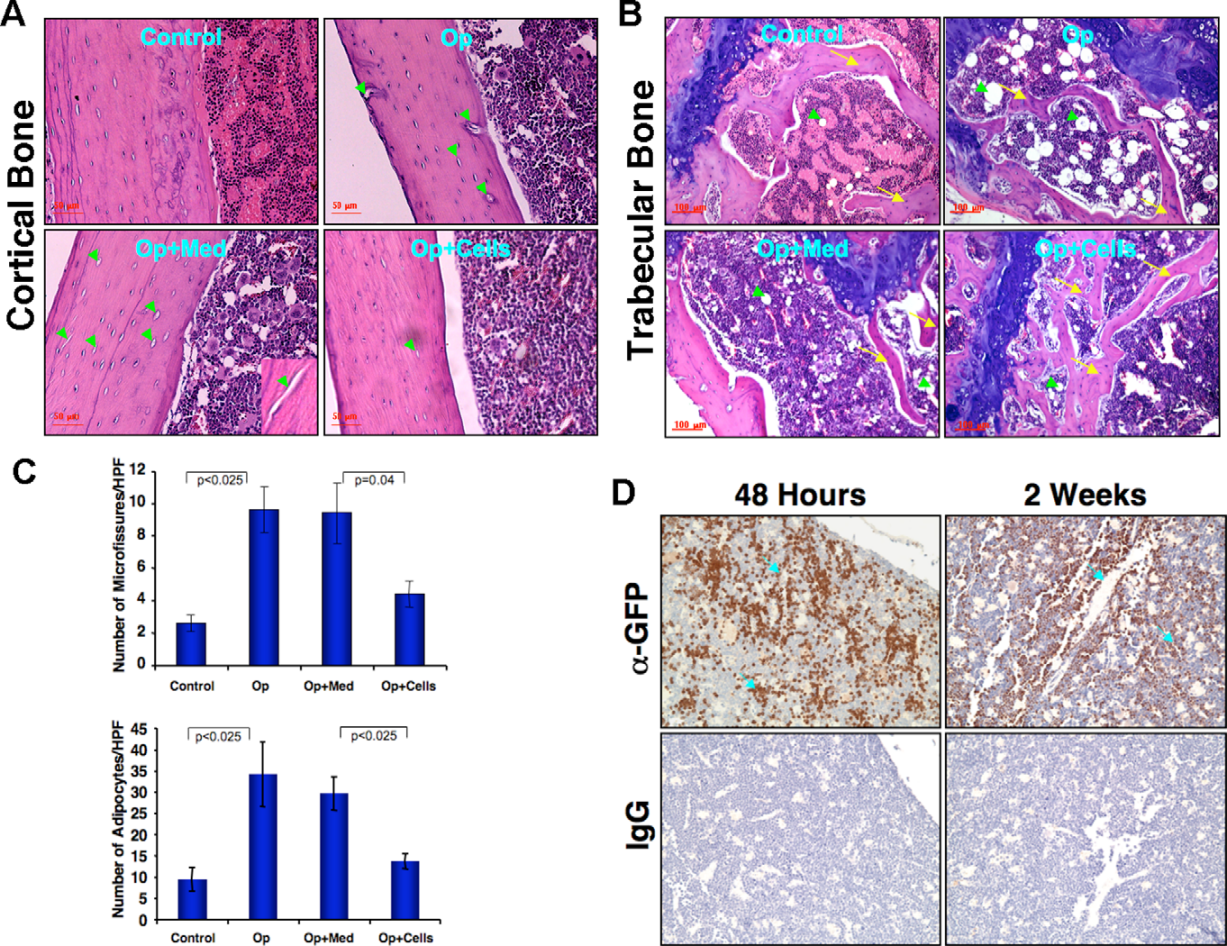
Effect of CD34^+^ cells on bone histomorphology in femurs of osteoporotic NOD/SCID mice. Osteoporosis (Op) was developed by injection of dexamethasone (for 21 days and 5 days for withdrawal) or saline as a control (Control) in seven month-old female NOD/SCID mice. Nanofiber-expanded CD34^+^ cells (half a million per mouse) were injected to the osteoporotic mice (Op+Cells) and serum-free medium was used as a media control (Op+Med). Femurs were harvested after 28 days of CD34+ cell injection, fixed, embed and H & E staining was performed. (**A**). An increased number of cortical bone micro fissures (arrowheads) were found in Op mice compared to control and numbers were reduced in Op+Cells animals. (**B**). A decreased number of trabecular bone spicules (arrows) and increased numbers of adipocytes (arrowheads) were found in Op mice compare to control under the growth plate region. In Op+Cells animals increased number of trabecular bone spicules and a decreased number of adipocyes were observed (n = 6/each group). (**C**). Evaluated numbers of microfissures and adipocytes per high-power field (HPF) in femur bone sections were shown in a graphical form (n = 6, four HPF/section). (**D**). Detection of systemically delivered GFP+ nanofiber-expanded human CD34^+^ cell in the bone marrow after 48 h and two weeks. Arrows indicate trabecular bone and sinusoids within the bone marrow.

We further investigated the therapeutic potential of nanofiber-expanded human CD34^+^ in a mouse model of dexamethasone-induced osteoporosis in immunocompromised NOD/SCID mice. Mice (n = 6/group) injected with dexamethasone for 21 consecutive days, followed by withdrawal for 5 days with tapering dose, and subsequently were either not treated and sacrificed (Op), treated with CD34^+^ cells via intra-cardio-ventricular injection (Op+ Cells; 0.5×10^6^/mouse), or treated with DMEM alone (Op+Med). Mice in all groups did not exhibit weight loss or gain during the course of experimentation. Hematoxylin and eosin (H & E) staining of longitudinal sections of femurs showed a decrease in the number of trabecular bone spicules in Op and Op+Med mice, as compared to untreated controls. However, after 28 days of CD34^+^ cell transplantation, an increase in the numbers of trabecular bone spicules was observed (Figure 2B, arrows). The number of adipocytes was significantly increased in Op and Op+Med mice, as compared to untreated control (Figure 2B, arrowheads). Further, the number of adipocytes was evaluated in each group and a marked reduction in adipocytes was observed in Op+Cells mice, as compared to Op mice. The number of adipocytes/high power field (HPF) was: control, 9.5±2.7; Op, 34.25±7.5; Op+Media, 29.75±3.9; Op+Cells, 13.75±1.7 (Figure 2C, lower panel).

### CD34^+^ cells home to the bone marrow

The chemokine receptor, CXCR4 binds to SDF-1, a chemotactic ligand expressed by bone marrow cells. Additionally, the presence of lymphocyte adhesion molecules (LFA-1) is required for the bone marrow homing of CD34^+^ cells. Since, we observed that both CXCR4 and LFA1 are highly expressed on CD34^+^ cells after 10 day of expansion on nanofibers, we next sought to examine the homing of these cells in the osteoporotic mice. Although delivered systemically, via intra-cardio-ventricular injection, CD34^+^ cells home to bone marrow (Figure 2D) as well as other organs such as lung, liver and spleen (data not shown). In the bone marrow, CD34^+^ were detected near the endosteal sites and around the bone marrow sinusoids, as well as at the surface of trabecular bone spicules after 48 hours (Figure 2D, upper left panel), as well as after 2 weeks (Figure 2D, upper right panel).

### Mineral apposition rate in response to CD34^+^ cell transplantation

**Figure 3:**
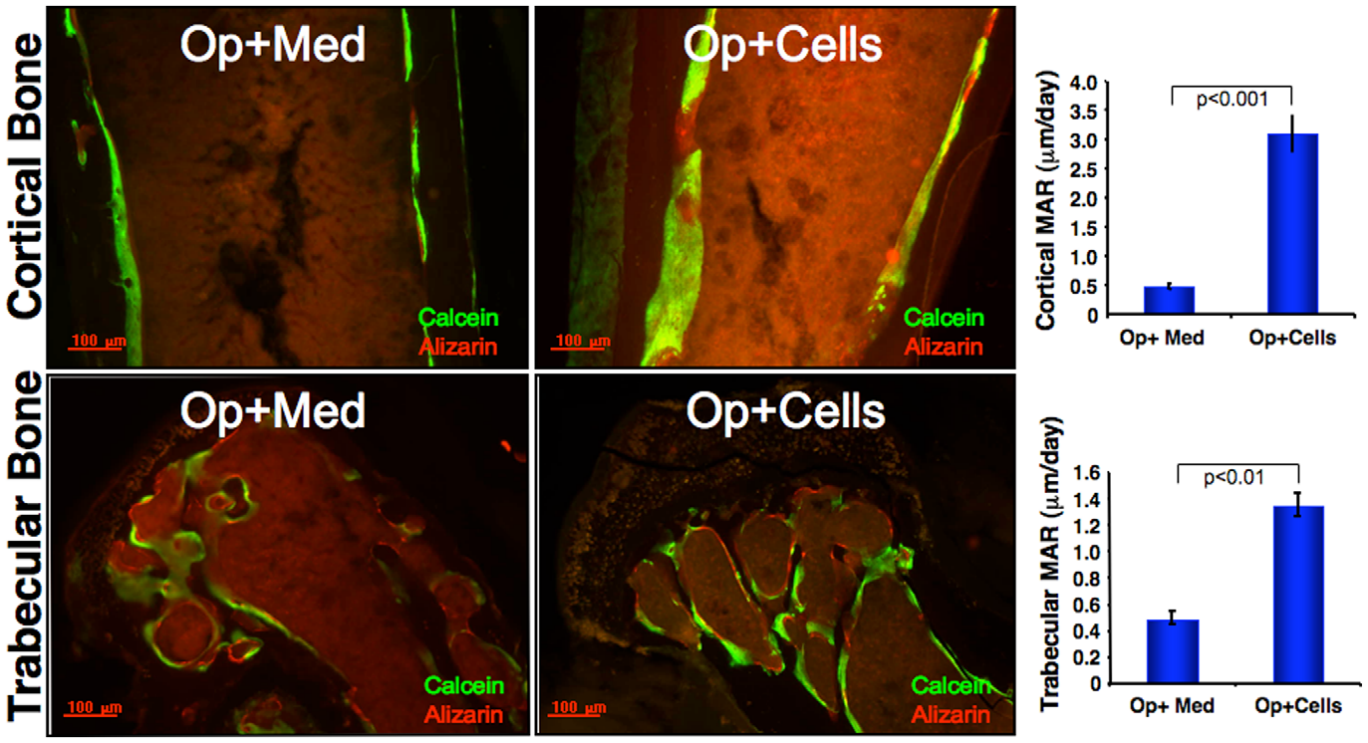
Detection of *in vivo* bone regeneration. *In vivo* immunolabeling with calcein (green) and alizarin (red) was performed in osteoporotic mice that received CD34^+^ cell (Op+Cells) or medium (Op+Media) as a control 10 and 3 days respectively before sacrifice of the mice. Femurs were harvested, formalin fixed and then embedded in methylmethacrylate resin. Thirty μm thick sections were mounted on the slides and observed under a fluorescence microscope. Increased mineral apposition rate (MAR) was observed in the endosteal sites of the cortical bone as well as trabeculae, distal from the growth plate, as detected by green fluorescence in mice that received CD34+ cells compared to control (n = 3/group). The values of MAR (μm/day) for the cortical and trabecular bone are shown graphically.

To determine the effect of CD34^+^cell transplantation on the mineral apposition rate (MAR), calcium binding fluorescent dye calcein (green) was injected at 17 days post CD34+ cell or medium injection. Subsequently, on day 24, fluorescent dye alizarin (red), and femurs were harvested on day 28. The inter-label distance between the two dyes was narrower at cortical and trabecular regions of the Op+Med mice compared to the Op+Cells mice indicating limited bone deposition in Op+Med mice. The values for mineral apposition rate were assessed in plastic embedded femur bone sections of Op+Med and Op+ Cells on the endosteal surface of metaphysial region of the femur, distal to the growth plate (Cortical MAR, μm/day; Op+Med, 0.47±0.04; Op+Cells, 3.07±0.29). Similarly, significant increase in trabecular MAR was observed in Op+Cells compared to Op+Med at the growth plate region (Trabecular MAR, µmm/day; Op+Med, 0.49±0.05; Op+Cells, 1.35±0.09) (Figure 3).

### Ultra structural analysis of bones after CD34^+^cell transplantation

**Figure 4:**
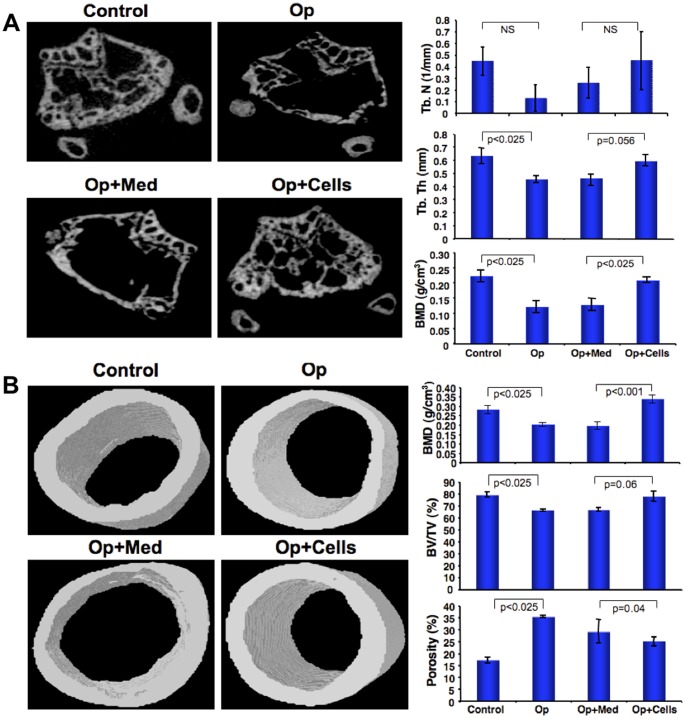
MicroCT images and analyses of bones. Formalin fixed femur bones were scanned using a high resolution MicroCT scanner (SkyScan 1172-D) established at 16 mm resolution and analyzed with the associated software (CTan). (**A**). Three dimensional image reconstruction of trabecular bones towards the distal side of the femur and, 0.1 mm away from the metaphyseal side of the growth plate is shown for each group (upper; left panel) and analysis is shown (upper; right panel) (n = 6/group). (**B**). MicroCT images (lower; left panel) and analyses of cortical bones (lower; right panel). Three dimensional (3D) image reconstruction of metaphyseal bones were generated at 2 mm away from growth plate and shown in the left panel. Analyzed data is presented in the lower; right panel (n = 6/group). NS =  non-significant.

To evaluate the extent of trabecular and cortical bone repair/regeneration and to image the differences in bone quality at the ultrastructural level, femurs from Op, Op+Med, Op+Cells were examined by micro computed tomography (MicroCT) (Figure 4A–1B, left panels). Quantitative analyses showed an increase in trabecular number in Op+Cells as compared to Op+Med mice (trabecular number, 1/mm; control, 0.46±0.1; Op, 0.11±0.1; Op+Med, 0.23±0.1; Op+Cells, 0.64±0.1) (Figure 4A, upper right panel). Similar trend was observed for trabecular thickness (mm): control, 0.63±0.06; Op, 0.45±0.02; Op+Med, 0.46±0.09; Op+Cells, 0.59±0.04. A significant increase in trabecular bone volume/ total volume was observed in Op+Cells mice, as compared to Op+Med mice, i.e., trabecular bone volume/total volume in control, 4.67±1.5; Op, 0.55±0.4; Op+Med, 1.59±1.1; Op+Cells, 10.22±3.8 (Figure 4A, lower right, panel). Similarly, similar pattern was observed for bone mineral density (BMD) of the trabeculae. BMD was significantly increased in Op+Cells mice compared to Op +Med (BMD, g/cm^3^; control, 0.223±0.02; Op, 0.121±0.02; Op+Med, 0.129±0.02; Op+Cells, 0.21±0.01). The reductions in BMD in Op mice indicated that dexamethasone treatments effectively decreased mineral density, and BMD was increased after CD34^+^ cell transplantation indicated reversal of the osteoporotic phenotype. Similarly, significant decrease in the degree of anisotropy (DA) was observed in the Op+Cells mice compared to Op+ Med mice (DA; control, 2.2±0.29; Op, 3±0.28; Op+Med, 2.6±0.23; Op+Cells, 1.75±0.1). Our data correlates with the previously reported results where higher degree of anisotropy was observed in osteoporotic bone compared to their healthy controls [22], [23]. Similarly, structure model index (SMI) of the trabeculae bone was reported to be an important predictor of changes in micro-architecture of trabeculae in osteoporotic conditions. SMI indicates three-dimensional shape of the trabecular bone. Value of SMI for ideal plate is 0 and for ideal rod is 3 [24]. Transition from plate to rod shape has been reported in osteoporotic and aged bones when compared to the healthy controls [25]. Similarly, our data showed a transition from more rod like structures in Op, Op+Med mice and more plate like in Op+Cells mice (SMI; control, 0.2±0.22; Op, 1.0 ±0.017; Op+Med, 1.13±0.25; Op+Cells, 0.27±0.08).

Metaphysial bones were also analyzed for cortical porosity, ratio of total bone volume to tissue volume and bone mineral density (BMD). MicroCT analysis of cortical bones revealed significant decrease in BMD in Op+Cells mice as compared to Op+Med mice, (BMD, g/cm^3^; control, 0.28±0.02; Op, 0.2±0.01; Op+Med, 0.19±0.02; Op+Cells, 0.33±0.01). Similar results were obtained for ratio of cortical bone volume/tissue volumes (control, 79.64±2.13; Op, 66.4±0.86; Op+Med, 67±1.34; Op+Cells, 77.9±4), suggesting a marked increase in cortical bone volume/tissue volume in Op+Cells mice as compared to Op+Med mice. Furthermore, results for cortical bone revealed a non-significant decrease in porosity of cortical bones in Op+Cells mice as compared to Op+Med mice, (cortical bone porosity (%); control, 17.17±1.3; Op, 35.52±0.5; Op+Med, 29.35±4.9; Op+Cells, 25.03±1.77) (Figure 4B, upper right panel).

### CD34^+^ cell transplantation elevated serum levels of osteocalcin

**Figure 5:**
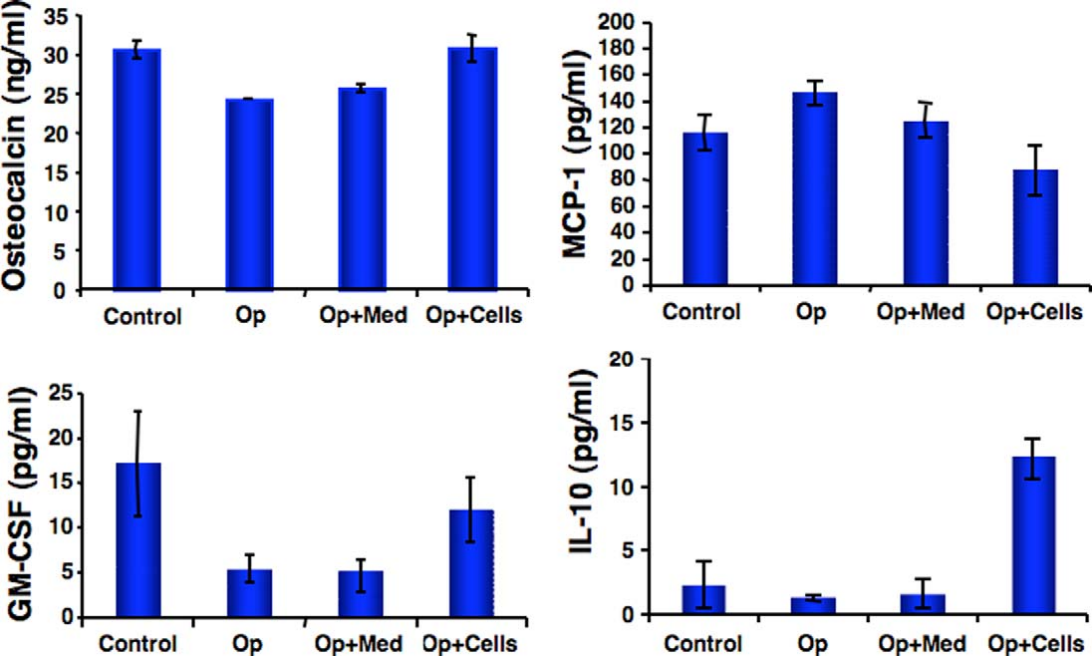
Serum levels of cytokines and growth factors. Blood was collected from all groups of animals (Control, Op, Op+Med and Op+Cells) before sacrifice and collected serum was stored at −80°C freezer. Increased levels of serum osteocalcin (indicative of bone formation) were observed in the mice that received CD34^+^ cell. Sandwich ELISA was performed to evaluate serum levels in all groups of animals using an osteoclacin ELISA kit (Biomedical Technologies, Inc, Staughton, MA), (n = 4 in triplicate). To assess the levels of various cytokines and growth factors (twenty factors) from collected serum (250 μl/animal) the multiplex ELISA was performed in triplicate by Quansys Biosciences, Logan Utah (n = 4/group). The values of MCP-1, GM-CSF and IL-10 were graphically reported as mean ± SEM.

As osteocalcin is the characteristic marker of osteoblast function, the levels of osteocalcin in the serum were evaluated to investigate *in vivo* effects of CD34+ cell therapy on osteoporotic mice. As shown in Figure 5, after osteoporosis, the serum levels of osteocalcin decreased as compared to untreated control mice. However, after CD34^+^cell transplantation, the levels of serum osteocalcin (ng/ml) were elevated, as follows: control, 30.5±1.1; Op, 24.2±0.04; Op+Med, 27.5± and Op+Cells, 30.72±1.7. This increase in the levels of serum osteocalcin indicates potential increase in the activation of osteoblasts and bone formation, as a consequence of CD34^+^cell transplantation in Op mice.

### Serum levels of cytokines and growth factors

It was reported that *in vitro* cultures of osteoblasts produce factors such as granulocyte-macrophage colony stimulating factor (GM-CSF) and interleukin-1 (IL-1) [26]. Multiplex ELISA was performed to analyze the levels of serum cytokines and growth factors implicated to play significant role in bone homeostasis (from Quansys Biosciences, Logan Utah). Out of 20 markers tested (n = 4 mice/group) four (MCP-1, GM-CSF, and IL-10) showed marked changes in all groups (Figure 5). CD34^+^ cell transplantation appeared to direct normalization of the levels of markers of the osteoporosis. GM-CSF and MCP-1 levels were shown to have opposite effects on the osteoclast function [14]. Our data revealed a marked increase in MCP-1 levels in the Op mice while MCP-1 levels plummet after the CD34^+^ cell transplantation in Op mice (MCP-1 in control, 116.77±7.1; Op, 156.92±12.8; Op+Med, 125.57±7.8; Op+Cells, 87.95±9.3). Reverse trend was observed for GM-CSF levels (control, 17.95±2.7; Op, 5.6±1.3; Op+Med, 5.1±1.5; Op+Cells, 12.22±1.7). Interestingly, we observed that levels of IL-10 (pg/ml) were suppressed in Op and Op+Med mice but were dramatically upregulated in Op+Cells mice (IL-10 in control, 2.925±0.8; Op, 1.3± 0.1; Op+Med, 1.975±0.5; Op+Cells, 12.5±2.3). As there was a high variation among the animals and number of animals was small, results were not statistically significant when posthoc analysis was performed by using Bonferroni correction.

### Impaired osteoclast differentiation following CD34^+^ cell transplantation

**Figure 6:**
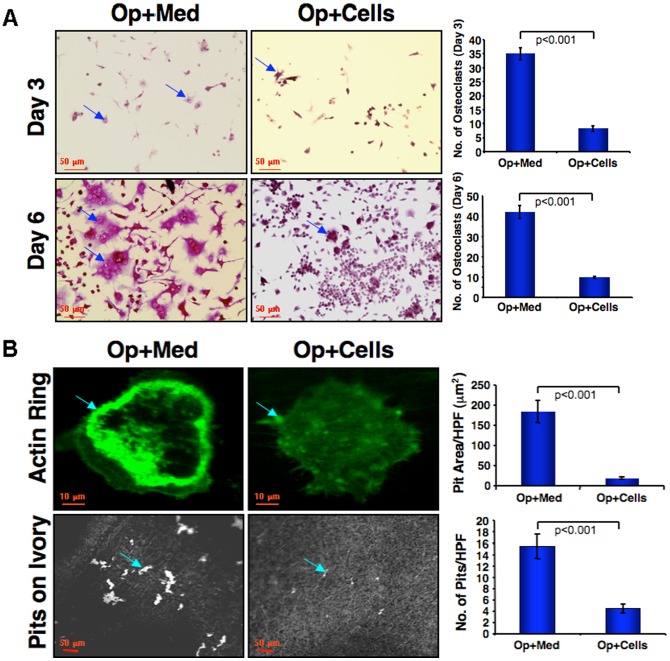
Impaired osteoclast differentiation, maturation and functionality in osteoporotic mice received CD34^+^ cells. Harvested bone marrow was subjected to differentiation towards osteoclasts using M-CSF and sRANKL (n = 5). (**A**). During the course of differentiation at day 3 and 6, cells were stained with tartrate-resistant acid phosphatase (TRAP). The purple color was considered to indicate TRAP+ cells (arrows, left panel) and the yellow color as TRAP- cells. Nuclei were stained with DAPI for counting cell numbers. The Op+Med showed an increased number of TRAP+ cells at day 3 (upper, right panel) as well as day 6 compared to Op+Cells (lower, right panel). Evaluated values of differentiated osteoclasts are graphically presented. (**B**). To determine osteoclast functionality on day 4 of differentiation, osteoclasts were harvested and plated on thin ivory slices. Bone resorption assays were performed using osteoclasts from all four groups of animals and to analyze the formation of F-actin rings on ivory bone slices (arrow, upper; left panel). Ivory slices were stained with hematoxylin and analyzed for the resorbed pits on day 10 of differentiation. The formation of F-actin rings was assessed by F-actin specific antibody (green fluorescence). F-actin rings were prominent in osteoclasts derived from Op+Med animals (arrow, upper; left panel), however, osteoclasts derived from Op+Cells animals stained negative for F-actin ring (arrow, upper; right panel). Fewer and smaller resorption pits formed by osteoclasts from Op+Cells animals (arrow, lower; right panel) compared to those formed by animals from Op+Med (arrow, lower; left panel). Evaluated values of pit area/ high power field (HPF) and number of pits/ high power field (HPF) are shown graphical.

Our observations regarding altered levels of cytokines and growth factors and increased bone formation suggested possible effects of CD34^+^ cell transplantation on differentiation and/or function of bone resorbing osteoclasts. Thus, we investigated any changes in differentiation or function of osteoclasts in Op mice with or without CD34^+^ cell transplantation. Bone marrows harvested from mice from all groups were allowed to adhere to the plastic overnight in the presence of M-CSF. Non-adherent cells were then induced to osteoclastic differentiation in the presence of M-CSF and RANKL. During the course of differentiation, cells were harvested at various time points (day 3 and 6) and osteoclast specific tartrate resistant acid phosphatase (TRAP) staining was performed to detect cells differentiating into mature osteoclasts. Osteoclasts were defined multinucleated TRAP+ cells. At day 3 and 6, significant numbers of cells were positive for TRAP staining in Op+Med mice. By day 6, extensive numbers of large multinucleated cells were positive for TRAP staining in the osteoporotic animals (Figure 6A, left panel). However, significantly less numbers of TRAP positive multinuclear cells were observed in animals that received CD34^+^ cells (Figure 6A, right panel). Quantification of TRAP+ cells at both days 3 and 6 is shown graphically (Figure 6A).

### Reduced functionality of osteoclasts after CD34^+^ cell transplantation

Osteoclasts use actin rich, ring-shaped attachment structures called sealing zone instead of focal adhesions for adherence to mineralized substrate. Generation of the sealing zone is requirement for osteoclasts to resorb bone. Therefore, to evaluate the osteoclast function, cells were immunostained for F-actin ring formation using anti-F-actin specific antibody. The cells from bone marrow of Op mice (Op+Med), demonstrated formation of clear F-actin rings (Figure 6B, upper left panel). Contrarily, clear F-actin ring in the bone marrow cells from Op+Cells mice was not observed. We next assessed whether F-actin ring lacking bone marrow cells from Op+Cells mice could resorb bone, using ivory slices as substrate. As shown in Figure 6B, lower right panel, osteoclast-like cells from Op+Med mice resorbed bone as evidenced by the large pits formed in ivory slices. The number and size of resorbed pits were significantly reduced in cells from Op +Cells mice (Figure 6B, lower left panel), indicating that CD34+ cell therapy significantly reduced osteoclast differentiation and function (number of pits/ HPF: Op+Med, 15.5±2.1; Op+Cells, 4.5±0.7; and pit area (μm^2^)/ HPF: Op+Med, 1420.26±329.3; Op+Cells, 164.3±19.6).

## Discussion

Despite the hematopoietic origin of nanofiber expanded CD34^+^ cells, herein we show that these cells could be differentiated towards osteogenic lineage. Both hematopoietic and osteogenic cells express similar families of transcription factors such as Cbfa/ Runx with some differences in expression levels of their sub family members [27], [28]. These observations led us to believe that hUCB derived CD34^+^ cells might also have potential to differentiate in the osteoblastic lineage. Indeed, we have observed that CD34^+^ cells in response to osteogenic signals, ascorbic acid and b-glycerophosphate, expressed Runx2, osteocalcin, osteonectin, collagen1A1, alkaline phosphatase, and BMP, Smad and Wnt signaling molecules, which are characteristically expressed by osteoblasts [29]. Runx2 is required for osteogenic differentiation of mesenchymal cells, whereas osteocalcin, osteonectin and collagen type1A1 are integral to bone matrix [9], [10], [30]. BMPs expressed by osteoblasts, bind and transduce signals via receptors to activate Smad 1/5/8 signaling pathway, to induce gene expression for bone-associated proteins [10]. Wnt signaling associated proteins Dvl2 and Dvl3 are required to transmit signals during osteoblastic differentiation [31], [32]. These findings provide evidence that hUCB derived CD34^+^ cells are multipotent in nature and microenvironmental cues could drive their differentiation towards osteoblastic lineages.

Characteristic hallmark of osteoporosis is the loss of bone. We have observed that, following dexamethasone treatment mice exhibit significant decreases in trabecular and cortical BMDs compared to controls. Trabecular bone micro architectural parameters such as anisotropy and structure model index also provide strong evidences that dexamethasone was able to induce osteoporosis. This bone loss and changes in bone micro-architecture were almost restored within 28 days following application of a single bolus of hUCB derived CD34^+^ cells. In osteoporosis, the balance of osteoblast and osteoclast cells is severely compromised. The number of osteoclasts is increased and they obliterate bone micro-architecture, which ultimately results in loss of bone mass [33], [34]. This is further accompanied by the bone marrow stromal cells differentiation towards adipogenesis and inhibition of osteoblastogenesis [35]. In the current study, we found the loss of trabecular bone spicules and loss of cortical bones in the osteoporotic mice and concurrent increment of adipocytes. However, application of nanofiber-expanded CD34^+^ cells induced significant reversion of osteoporosis, by increment of bone formation presumably by osteoblasts and concomitant decrease in number of adipocytes and osteoclasts. In fact, circulating CD34^+^ cells have been shown to be recruited to the skeletal defect sites and heal non-union fractures in murine models [36], [37]. The chemokine receptor, CXCR4 binds to stromal derived factor (SDF)-1 expressed highly in bone marrow stromal cells, and is required for efficient homing of cells to the bone marrow [38]. Since, nanofiber-expanded CD34^+^ cells constitutively express high levels of CXCR4, this may be critical for homing of these cells to bone marrow. In a preclinical model of osteoporosis, adenoviral-mediated overexpression of CXCR4 gene was shown to be necessary for bone marrow derived mesenchymal cells, to home in the bone marrow [39]. However, advantages of using the nanofiber-expanded cells are that we do not need to induce CXCR4 expression using any viral methods, which has potential to integrate to the host genome [40]. Although nanofiber-expanded cells home to the other organs such as lung or spleen besides bone marrow, no bone formation was observed in other organs. Also, recently it was shown that CXCR4 expression on the precursor cells was important for bone formation [41]. Additionally, hUCB derived CD34^+^ cells have greater advantage in that these cells have not been reported to induce oncogenic transformation in experimental model systems [20].

During osteoporosis, osteoblasts undergo apoptosis, thereby, altering the bone formation and lowering bone mineral density. This is paralleled by lower levels of serum osteoclacin, a marker for bone turnover [42]. Current findings show that CD34^+^ cell transplantation decreased the numbers of adipocytes in bone marrow, increased trabecular numbers and thickness, and increased BMD, thereby indicating induction of osteoblast in bone. An increase in the osteocalcin levels coupled with an increase *in vivo* mineral apposition rate in the mice that received CD34^+^ cell transplantation, confirms the regenerative potential of CD34^+^ cells in bone formation.

Elevated levels of GM-CSF, and IL-10 and decreased level of MCP-1 in serum were also evident with the CD34^+^ cell transplantation. Previous *in vitro* studies have shown that in the presence of CD34^+^ cells, osteoblasts secrete GM-CSF and cytokines such as IL-6, IL-4 and IL-1 involved in bone homeostasis [15], [43], [44]. Current findings suggest that CD34^+^ cells likely modulate cytokine secretions during induction of osteogenesis. In this respect, we have observed high level of IL-10 in serum of the mice that received CD34^+^ cells. The importance of IL-10 in bone metabolism was demonstrated in IL-10 deficient mice, which develop osteopenia and decreased bone formation [45], [46]. IL-10 exhibits anti-osteoclastic activity and may directly inhibit osteoclast precursor cell differentiation [47]. CD34^+^ cell transplantation thus acts by upregulating the levels of IL-10 that may in turn regulate the bone remodeling by impairing osteoclastogenesis and restoring the bone formation [46], [47].

Above findings thus demonstrate a novel therapeutic potential of nanofiber-expanded CD34^+^ cells in resolving osteoporosis. These cells provide several advantages over other cell types as they are easily accessible, less immunogenic and maintain multipotency. They can be available in unlimited numbers via expansion on nanofibers; constitutively express CXCR4 for efficient homing to the bone marrow; and differentiate into osteogenic lineage *in vitro*. These cells are biologically functional as they significantly improve bone mineral density, bone formation and bone micro-architecture in osteoporotic murine model. These characteristics make them an attractive choice of cells for bone induction in patients with severe bone loss due to osteoporosis.

## Materials and Methods

### Isolation of CD133^+^ hematopoietic cells

Fresh human umbilical cord blood was obtained from The Ohio State University Medical Center with written consent from donors and prior approval from the internal review board. Briefly, heparinized cord blood was diluted with PBS (1∶1) and carefully layered over 10 ml of Ficoll-Paque (GE Health Care-Biosciences, USA). After 30-min centrifugation in a swinging bucket rotor at 1400 rpm, the upper layer was aspirated and the mononuclear cell layer (buffy coat) was collected. Following labeling with magnetic bead conjugated anti-CD133 monoclonal antibody (Miltenyi Biotec Inc, Bergisch Gladbach, Germany), two cell separation cycles (with different columns) were performed using the AutoMACS cell sorter (Miltenyi Biotec) following manufacturer's protocol and reagents. After separation, purity of the cell product was determined by flow cytometry (more than 95% cells were CD133^+^). Total 4–6 samples of cord blood were procured, isolated and used nanofiber mediated expansion and *in vitro* and *in vivo* experiments.

### Expansion of CD133^+^ hematopoietic cells

Electrospinning, surface grafting, and amination of PES nanofibers were carried out according to the procedure described earlier [48]. All chemicals were purchased from Sigma-Aldrich (USA) unless otherwise stated. PES granules were purchased from Goodfellow Cambridge Limited, UK. CD133^+^ cells were expanded on nanofiber-coated plates using serum free expansion medium (SFEM) as previously described [19]. In brief, purified recombinant human stem cell factor (SCF), Flt-3 ligand (Flt3), thrombopoietin (TPO), and IL-3 were purchased from Peprotech Inc. (Rocky Hill, NJ). The StemSpan SFEM medium was purchased from StemCell Technologies (Vancouver, BC, Canada). Nanofiber meshes were securely glued to the bottoms of wells of a 24-well tissue culture plate. Eight hundred CD133^+^ cells were seeded onto each scaffold in 0.6 ml StemSpanTM serum-free expansion medium, which consists of 1% BSA, 0.01 mg/ml recombinant human insulin, 0.2 mg/ml human transferrin, 0.1 mM 2-mercaptoethanol, and 2 mM L-glutamine in Iscove's MDM, supplemented with 0.04 mg/ml low-density lipoprotein (Athens Research and Technology Inc., USA), 100 ng/ml SCF, 100 ng/ml Flt3, 50 ng/ml TPO, and 20 ng/ml IL-3. Cells were cultured at 37°C in an atmosphere containing 5% CO_2_ for 10 days without medium change. Cells were harvested after 10 days of expansion. All wells were washed once with non-enzymatic cell dissociation solution and twice with 2% FBS containing Hanks' buffer at 5 min intervals between each wash. The collected cells were then concentrated through centrifugation at 300× g for 5 min. Aliquots of the cells were then used for cell counting by a hemocytometer, flowcytometric analysis, as well as for further studies.

### Flowcytometry

Flowcytometric analysis was performed by using standard two colors staining with a FACS Calibur flowcytometer (Becton Dickinson, Heidelberg, Germany) as described earlier [19]. Non-specific Fc-receptors were blocked with FcR-blocking reagent (Miltenyi Biotec Inc.) prior to adding primary antibodies. Primary antibody was incubated for 30 min at 4°C with the aliquots of expanded cells. Antibodies used were anti-CD34-PE, anti-CD133/2-FITC (all from Miltenyi Biotec Inc), PE labeled CXCR4, von Willebrand Factor, CD31, CD45, MHC class I, MHC class II, CD69, CD3, Mac-I, LFA-1, CD86, CD14 and isotype controls were purchased from BD Biosciences (USA) After incubation cells were washed with MACS buffer and resuspended in MACS buffer. Dead cells were excluded via propidium iodide staining. Data analysis was performed with BD Cell Quest software. The Milan-Mulhouse gating method was used for cell enumeration, where a double gating (CD133^+^ and CD34^+^) strategy was used to identify the primitive hematopoietic progenitor cell populations. At least 20,000 events were acquired.

### Osteoblastic differentiation of nanofiber-expanded CD34^+^ cells

After 10 days of CD133^+^ cell expansion on nanofiber, cells were collected and reseeded in DMEM supplemented with 10% fetal bovine serum (FBS), penicillin, streptomycin, and glutamate (PSG) for three days in a 24-well plate or 4-chamber slides (Lab-Tek II Chamber slide System, Nalge Nunc International Corp., Naperville, IL, USA). The cultured cells were induced by specific osteoblast differentiating factors (60 μM ascorbic acid and 10 mM β-glycerophosphate) dissolved in fresh DMEM complete medium. The differentiation process was continued for 3 weeks with a change of fresh medium containing specific osteoblast inducing factors in every 3rd day. During the process of differentiation, some of the wells of differentiated cells were harvested at specific time points for further studies (n = 4).

### Alizarin red S staining

To examine *in vitro* mineralization in osteoblastic differentiated cells, Alizarin Red S staining was performed in wells of a 24-well plate. Briefly, after 21 days of differentiation cells were fixed in 70% ethanol for one hour at room temperature followed by a wash with water (10 minutes, 2 times) and incubated with 1% Alizarin Red S solution for 30 minutes at room temperature (Chemicon International, USA). The red stain was washed with water three times and cells were mounted with mounting solution. Images were obtained by using a digital camera attached to the microscope (Axioplan2; Carl Zeiss) using Axio-vision software (Carl Zeiss, NY, USA). Nanofiber-expanded CD34^+^ cells cultured in DMEM complete medium were used as a control.

### Immunocytochemical staining

One quarter of a million nanofiber-expanded CD34^+^ cells were induced to differentiate in each well of 4-chamber slides for three weeks. After three weeks of culture immunocytochemistry was performed following standard protocol to assess osteoblastic differentiation using osteoblast specific markers such as Smad 1/5/8, osteocalcin and IgG as an isotype control. Nuclear stain was performed with DAPI. Slides were observed under a fluorescence microscope (Axioplan2; Carl Zeiss) and images were captured with Zeiss Axiovision imaging software (Carl Zeiss, NY, USA).

### RT-PCR and western blot analyses

Nanofiber-expanded CD34^+^ cells were induced to osteoblastic differentiation *in vitro*. During the course of osteoblastic differentiation, total RNA and proteins were isolated at various time points using RNeasy Kit (Qiagen, USA) and RIPA, cell lysis buffer respectively. One microgram of RNA was used for cDNA synthesis using oligo dT (Invitrogen, USA) primer. Semi quantitative PCR was performed using one micro liter of cDNA for the gene specific primers such as Runx2, alkaline phosphatase, osteocalcin, osteonectin, collagen 1A1 and GAPDH. Fold increase for expression of each gene at mRNA level was analyzed using UN-SCAN-IT software (Silk Scientific, Inc.). Nanofiber-expanded cells were used as a control. Primers for RT-PCR reactions were designed using Primer Blast-NCBI software: Human Runx2 forward primer: 5′ TAAGTACACGGGCTTCAGGG 3′; Human Runx2 reverse primer: 5′ TTGTTGTCTTCTTGCCTCCA 3′; Human Alk Phos forward primer: 5′ GGACATGCAGTACGAGCTGA 3′; Human Alk Phos reverse primer: 5′ CACCAAATGTGAAGACGTGG 3′; Human osteocalcin forward primer:5′ AAGCAAGTAGCGCCAATCT 3′; Human osteocalcin reverse primer: 5′ GGAAGTAGGGTGCCATAACAC 3′; Human osteonectin forward primer:5′ ACATCGGGCCTTGCAAATACA 3′; Human osteonectin reverse primer: 5′ GAAGCAGCCGGCCCACTCATC 3′; Human collagen1A1 forward primer:5′ CCTGGCCCCATTGGTAATGTT 3′; Human collagen1A1 reverse primer: 5′ CCCCCTCACGTCCAGATTCAC 3′; Human GAPDH forward primer: 5′ CTGATGCCCCCATGTTCGTC 3′; Human GAPDH reverse primer: 5′ CACCCTGTTGCTGTAGCCAAATTCG 3′.

Similarly, Western blot was performed for the level of BMP2, BMP4, Smad-1/5/8 (all from Santa Cruz Biotechnology, Inc., CA), Dvl2, Dvl3, and GAPDH (all from Cell Signaling Technology, Inc. MA) protein. Nanofiber-expanded cells were used as a control. Induced differentiated MC3T3 cells were also used as a positive control. Fold increase for expression of each gene at protein level was analyzed using UN-SCAN-IT software (Silk Scientific, Inc.).

### Generation of osteoporosis

Seven month-old female NOD/SCID mice, retired breeder (body weight approximately 25 g) were obtained from Jackson Laboratory, Bar Harbor, ME and used for osteoporosis induction. The mice were housed in sterile IACUC-approved facilities at the Biomedical Research Tower at The Ohio State University. After a week of acclimatization, mice were intraperitoneally (i.p.) injected with 5 mg/ kg body weight (b. wt.) of dexamethasone (American Regent, Inc. Shirley, NY) or with saline (as a control) for consecutive 21 days as described before [35]. Tapering doses of dexamethasone were given for 5 days for withdrawal. The mice were weighed every week during the injection to record any significant weight loss.

### CD34+ cell transplantation

The mice, that received glucocorticoid injections were either sacrificed after 26 (21+5) days as an osteoporotic control or were used for CD34+ cell / medium injections (6 mice/group). The osteoporotic mice were anesthetized and injected with nanofiber-expanded CD34+ cells (0.5 million in 300 μl of serum free medium) via intra-cardio-ventricular injection or 300 μl of serum free medium (Op+Med) as a control for CD34+ cell therapy group. The osteoporotic mice of CD34+ cell therapy (Op+Cells) group received only one dose of CD34+ cells and were sacrificed after 28 days of injection. Osteoporotic mice receiving medium (Op+Med) were also sacrificed at the same time point.

### CD34^+^ cell homing

To assess the homing of CD34+ cells, GFP overexpressed nanofiber-expanded CD34^+^ cells (0.5 million cells/mouse) were injected into the mice via intra-cardio-ventricular injection after induction of osteoporosis. Forty-eight hours and two weeks after cell injection, mice were sacrificed and various organs were harvested. Tissues were fixed in 10% formalin solution and subsequently embedded in paraffin block. Five-micron sections were cut and stained using anti-GFP primary antibody (Zymed Laboratories, Inc, CA, USA) and detected with 3,3′-Diaminobenzidine (DAB) and mounted. Slides were observed under a microscope (Axioplan2; Carl Zeiss) and images were captured with Zeiss, Axiovision imaging software (Carl Zeiss).

### Mineral apposition rate (MAR)

A separate set of seven months old NOD/ SCID mice (3 mice /group) were subjected to generation of osteoporosis (21+5 days) and followed by CD34^+^ cell transplantation or medium (as a control) for 28 days as described above. Mice were intraperitoneally (i.p.) injected with calcium binding dye, calcein (green flourescent dye; 10 mg/kg b.wt., Sigma); 10 days prior to sacrifice. Alizarin Red Dye (red fluorescent dye; 50 mg/kg b.wt., Sigma) was also injected to the same mice via i.p. route three days prior to sacrifice. Upon sacrifice, femurs were harvested and formalin fixed for 24 hours and then washed with 1x PBS, dehydrated with 70% ethanol, 95% ethanol, 100% ethanol, infiltrated and embedded in methylmethacrylate and polyester resin. Thirty-micrometer thick sections were cut with a diamond blade using Saw Microtome Leica SP1600 (Leica Microsystem, Wetzlar, Germany). The sections were mounted on the slides using Vecta Mount (Vector Labs, Inc. Burlingame, CA) and observed under a fluorescence microscope (Carl Zeiss, NY, USA) and images were captured with Zeiss Axiovision imaging software (Axioplan2). Images were analyzed to assess the mineral apposition rate (MAR) by using NIH Image J software. The mineral apposition rate (MAR, in μm per day) was determined by dividing the mean width of the double labels by the inter-label time as described previously [49].

### Hematoxylin and eosin staining

All mice were euthanized after collecting blood and urine, and femurs were removed by surgical dissection and were preserved in 10% neutral buffered formalin. After decalcification, paraffin-blocked tissues were sectioned and stained with standard hematoxylin and eosin (H&E) staining protocol at the same depth from the growth plate.

### Micro computed tomography (MicroCT)

Formalin fixed femur bones were encased in a tight fitted plastic tube to prevent any motion during scanning. Femurs were scanned using a high-resolution Micro CT scanner (SkyScan1172-D, Kontich, Belgium) at 16μm resolution. For measurements of bone mineral density (BMD; g/cm^3^), phantoms of 25 and 75 g/cm^3^ were scanned with the same settings as applied to the mice femur bones from all groups. The scanned images were reconstructed using Skyscan Nrecon software and analyzed with the CTan software (Kontich, Belgium). Analyses of trabecular bone were carried out in distal femur, 0.1 mm away from the growth plate. A threshold was established and the same threshold value was kept constant for all samples. Similarly, the metaphysial region, 2mm away from the growth plate, was selected for the cortical bone analysis. A separate threshold was established and kept constant for all cortical bone measurements. The analyses were performed for the bone volume, bone to tissue volume ratios, trabecular number (Tb.N; 1/mm), trabecular thickness (Tb.Th; mm), trabecular bone mineral density (BMD; g/cm^3^), degree of anisotropy (DA), structure model index (SMI), cortical porosities (%), ratio of bone volume/ tissue volume (BV/TV) and cortical bone mineral density (BMD; g/cm^3^) in animals from all groups using the Skyscan software and following manufacturer's protocol. The results were reported in mean ± SEM (n = 6/ per group). Three-dimensional (3D) models were reconstructed using CTvol software from SkyScan.

### Serum osteocalcin assay

Sandwich ELISA was performed for the collected mouse serum using the osteocalcin ELISA kit (Biomedical Technologies, Inc, Staughton, MA). Briefly, the mouse was anesthetized and blood was collected from the descending aorta in an eppendorf tube and kept at room temperature for an hour to coagulate, then centrifuged at 14000 rpm for 20 minutes at 4°C. Serum was collected and stored at −80°C until further use. Standard curve was made and the serum samples were run in triplicates to assess the amount of osteocalcin in the serum. The samples from each group were diluted (5x) and incubated with the anti-serum osteocalcin overnight and detected using streptavidin-horseradish peroxidase (HRP) detecting system. The values were reported in mean ± SEM (n = 4/ per group, in triplicate).

### Multiplex ELISA for cytokines and growth factors

To assess the levels of various cytokines and growth factors (twenty factors from 250 μl of serum) from collected serum (from all groups of animals before sacrifice and stored at −80°C), the multiplex ELISA was performed in triplicate by Quansys Biosciences, Logan Utah (n = 4/ per group). The values were reported in mean ± SEM.

### Osteoclast differentiation

To assess the therapeutic effects of CD34^+^ cells on the bone resorbing cells; bone marrow cells were collected from femurs of animals of all the groups, after termination of experiments and were induced for osteoclastic differentiation *in vitro*. Cells were cultured overnight at 37°C incubator with 5% CO_2_ in αMEM containing 10% heat inactivated fetal bovine serum in the presence of 20 ng/ml M-CSF (R & D Systems, Minneapolis, MN). The next day, same number of non-adherent cells (1.5 million for all the groups) were collected and incubated for an additional 5–8 days in αMEM medium with 20 ng/ml M-CSF, and 50 ng/ml GST-RANKL [50]. The fresh medium was replaced every third day. At day 3 and 6 of differentiation, the cells were stained for TRAP staining using an acid phosphatase, leukocyte; TRAP staining kit (Sigma Aldrich, USA) and was viewed and imaged with a fluorescence microscope (Carl Zeiss, NY, USA). TRAP-positive cells (purple) containing at least three nuclei were counted as osteoclasts.

### Osteoclast cytoskeleton structure and functionality

Osteoclasts were generated on plastic dishes as described above and on 3^rd^ day of differentiation, osteoclasts cells were removed and equal number of osteoclasts cells were re-plated either on thinly cut ivory slices or glass cover slips. Cells were fixed at various time points of culture with 1% formaldehyde in pH 6.5 (30 minutes at room temp), stabilization buffer (127 mM NaCl, 5 mM KCl, 1.1 mM NaH_2_PO_4_, 0.4 mM KH_2_PO_4_, 2 mM MgCl_2_, 5.5 mM glucose, 1 mM EGTA, 20 mM Pipes), and subsequently fixed and permeabilized with 2% formaldehyde, 0.2% Triton X-100, and 0.5% deoxycholate in the same stabilization buffer. Cells were stained with F-actin specific Ab and visualized using a Zeiss 510 META laser scanning confocal microscope (Campus Microscopy and Imaging Facility, The Ohio State University). Actin ring and podosome thicknesses were determined by generating Z-stack images of randomly selected cells and these structures were measured at their thickest points [50]. Bone resorption was assessed using ivory slices and osteoclasts were gently removed with cotton swabs and washed with water. The ivory slices were then stained with hematoxylin stain for 5 minutes at room temperature and excess stain was removed by washing with water and pits were imaged with a confocal microscope mentioned above.

### Statistical analysis

Values were expressed as mean ± SEM and statistical analysis was performed by using JMP software (version 9, SAS Institute Inc. NC). After checking equal variance two sample ‘t’-test was performed for assessment of significance. Posthoc analysis was performed by using Bonferroni correction and significance was determined when p values were obtained less than 0.025.

## References

[1] Cooper C CG, Melton LJ 3rd (1992). Hip fractures in the elderly: a world-wide projection.. Osteoporos International.

[2] Aguila HL, Rowe DW (2005). Skeletal development, bone remodeling, and hematopoiesis.. Immunol Rev.

[3] Zaidi M (2007). Skeletal remodeling in health and disease.. Nat Med.

[4] Weinstein RS, Jilka RL, Parfitt AM, Manolagas SC (1998). Inhibition of osteoblastogenesis and promotion of apoptosis of osteoblasts and osteocytes by glucocorticoids. Potential mechanisms of their deleterious effects on bone.. J Clin Invest.

[5] Seeman E, Delmas PD (2006). Bone quality–the material and structural basis of bone strength and fragility.. N Engl J Med.

[6] Lecka-Czernik B, Gubrij I, Moerman EJ, Kajkenova O, Lipschitz DA (1999). Inhibition of Osf2/Cbfa1 expression and terminal osteoblast differentiation by PPARgamma2.. J Cell Biochem.

[7] Takada I, Kouzmenko AP, Kato S (2009). Wnt and PPARgamma signaling in osteoblastogenesis and adipogenesis.. Nat Rev Rheumatol.

[8] Pittenger MF, Mackay AM, Beck SC, Jaiswal RK, Douglas R (1999). Multilineage potential of adult human mesenchymal stem cells.. Science.

[9] Ducy P, Karsenty G (1995). Two distinct osteoblast-specific cis-acting elements control expression of a mouse osteocalcin gene.. Mol Cell Biol.

[10] Ducy P, Zhang R, Geoffroy V, Ridall AL, Karsenty G (1997). Osf2/Cbfa1: a transcriptional activator of osteoblast differentiation.. Cell.

[11] Phimphilai M, Zhao Z, Boules H, Roca H, Franceschi RT (2006). BMP signaling is required for RUNX2-dependent induction of the osteoblast phenotype.. J Bone Miner Res.

[12] Rawadi G, Vayssiere B, Dunn F, Baron R, Roman-Roman S (2003). BMP-2 controls alkaline phosphatase expression and osteoblast mineralization by a Wnt autocrine loop.. J Bone Miner Res.

[13] Kobayashi H, Gao Y, Ueta C, Yamaguchi A, Komori T (2000). Multilineage differentiation of Cbfa1-deficient calvarial cells in vitro.. Biochem Biophys Res Commun.

[14] Kim MS, Day CJ, Morrison NA (2005). MCP-1 is induced by receptor activator of nuclear factor-{kappa}B ligand, promotes human osteoclast fusion, and rescues granulocyte macrophage colony-stimulating factor suppression of osteoclast formation.. J Biol Chem.

[15] Marie PJ, Hott M, Launay JM, Graulet AM, Gueris J (1993). In vitro production of cytokines by bone surface-derived osteoblastic cells in normal and osteoporotic postmenopausal women: relationship with cell proliferation.. J Clin Endocrinol Metab.

[16] Vahle JL, Sato M, Long GG, Young JK, Francis PC (2002). Skeletal changes in rats given daily subcutaneous injections of recombinant human parathyroid hormone (1–34) for 2 years and relevance to human safety.. Toxicol Pathol.

[17] Wilting I, de Vries F, Thio BM, Cooper C, Heerdink ER (2007). Lithium use and the risk of fractures.. Bone.

[18] Khosla S, Westendorf JJ, Oursler MJ (2008). Building bone to reverse osteoporosis and repair fractures.. J Clin Invest.

[19] Das H, Abdulhameed N, Joseph M, Sakthivel R, Mao HQ (2009). Ex vivo nanofiber expansion and genetic modification of human cord blood-derived progenitor/stem cells enhances vasculogenesis.. Cell Transplant.

[20] Das H, George JC, Joseph M, Das M, Abdulhameed N (2009). Stem cell therapy with overexpressed VEGF and PDGF genes improves cardiac function in a rat infarct model.. PLoS One.

[21] Mifune Y, Matsumoto T, Kawamoto A, Kuroda R, Shoji T (2008). Local delivery of granulocyte colony stimulating factor-mobilized CD34-positive progenitor cells using bioscaffold for modality of unhealing bone fracture.. Stem Cells.

[22] Chappard C, Brunet-Imbault B, Lemineur G, Giraudeau B, Basillais A (2005). Anisotropy changes in post-menopausal osteoporosis: characterization by a new index applied to trabecular bone radiographic images.. Osteoporos Int.

[23] Ciarelli TE, Fyhrie DP, Schaffler MB, Goldstein SA (2000). Variations in three-dimensional cancellous bone architecture of the proximal femur in female hip fractures and in controls.. J Bone Miner Res.

[24] Borah B, Dufresne TE, Cockman MD, Gross GJ, Sod EW (2000). Evaluation of changes in trabecular bone architecture and mechanical properties of minipig vertebrae by three-dimensional magnetic resonance microimaging and finite element modeling.. J Bone Miner Res.

[25] Hildebrand T, Ruegsegger P (1997). Quantification of Bone Microarchitecture with the Structure Model Index.. Comput Methods Biomech Biomed Engin.

[26] Taichman RS, Emerson SG (1996). Human osteosarcoma cell lines MG-63 and SaOS-2 produce G-CSF and GM-CSF: identification and partial characterization of cell-associated isoforms.. Exp Hematol.

[27] Banerjee C, McCabe LR, Choi JY, Hiebert SW, Stein JL (1997). Runt homology domain proteins in osteoblast differentiation: AML3/CBFA1 is a major component of a bone-specific complex.. J Cell Biochem.

[28] Zeng C, van Wijnen AJ, Stein JL, Meyers S, Sun W (1997). Identification of a nuclear matrix targeting signal in the leukemia and bone-related AML/CBF-alpha transcription factors.. Proc Natl Acad Sci U S A.

[29] Beck GR, Zerler B, Moran E (2000). Phosphate is a specific signal for induction of osteopontin gene expression.. Proc Natl Acad Sci U S A.

[30] Xiao G, Cui Y, Ducy P, Karsenty G, Franceschi RT (1997). Ascorbic acid-dependent activation of the osteocalcin promoter in MC3T3-E1 preosteoblasts: requirement for collagen matrix synthesis and the presence of an intact OSE2 sequence.. Mol Endocrinol.

[31] Qiang YW, Hu B, Chen Y, Zhong Y, Shi B (2009). Bortezomib induces osteoblast differentiation via Wnt-independent activation of beta-catenin/TCF signaling.. Blood.

[32] Day TF, Guo X, Garrett-Beal L, Yang Y (2005). Wnt/beta-catenin signaling in mesenchymal progenitors controls osteoblast and chondrocyte differentiation during vertebrate skeletogenesis.. Dev Cell.

[33] Akune T, Ohba S, Kamekura S, Yamaguchi M, Chung UI (2004). PPARgamma insufficiency enhances osteogenesis through osteoblast formation from bone marrow progenitors.. J Clin Invest.

[34] Hong JH, Hwang ES, McManus MT, Amsterdam A, Tian Y (2005). TAZ, a transcriptional modulator of mesenchymal stem cell differentiation.. Science.

[35] McLaughlin F, Mackintosh J, Hayes BP, McLaren A, Uings IJ (2002). Glucocorticoid-induced osteopenia in the mouse as assessed by histomorphometry, microcomputed tomography, and biochemical markers.. Bone.

[36] Matsumoto T, Kawamoto A, Kuroda R, Ishikawa M, Mifune Y (2006). Therapeutic potential of vasculogenesis and osteogenesis promoted by peripheral blood CD34-positive cells for functional bone healing.. Am J Pathol.

[37] Matsumoto T, Mifune Y, Kawamoto A, Kuroda R, Shoji T (2008). Fracture induced mobilization and incorporation of bone marrow-derived endothelial progenitor cells for bone healing.. J Cell Physiol.

[38] Yu X, Huang Y, Collin-Osdoby P, Osdoby P (2003). Stromal cell-derived factor-1 (SDF-1) recruits osteoclast precursors by inducing chemotaxis, matrix metalloproteinase-9 (MMP-9) activity, and collagen transmigration.. J Bone Miner Res.

[39] Lien CY, Chih-Yuan Ho K, Lee OK, Blunn GW, Su Y (2009). Restoration of bone mass and strength in glucocorticoid-treated mice by systemic transplantation of CXCR4 and cbfa-1 co-expressing mesenchymal stem cells.. J Bone Miner Res.

[40] Nakai H, Montini E, Fuess S, Storm TA, Grompe M (2003). AAV serotype 2 vectors preferentially integrate into active genes in mice.. Nat Genet.

[41] Zhu W, Liang G, Huang Z, Doty SB, Boskey AL (2011). Conditional inactivation of the CXCR4 receptor in osteoprecursors reduces postnatal bone formation due to impaired osteoblast development.. J Biol Chem.

[42] Godschalk MF, Downs RW (1988). Effect of short-term glucocorticoids on serum osteocalcin in healthy young men.. J Bone Miner Res.

[43] Taichman RS, Emerson SG (1994). Human osteoblasts support hematopoiesis through the production of granulocyte colony-stimulating factor.. J Exp Med.

[44] Taichman RS, Reilly MJ, Verma RS, Emerson SG (1997). Augmented production of interleukin-6 by normal human osteoblasts in response to CD34+ hematopoietic bone marrow cells in vitro.. Blood.

[45] Al-Rasheed A, Scheerens H, Rennick DM, Fletcher HM, Tatakis DN (2003). Accelerated alveolar bone loss in mice lacking interleukin-10.. J Dent Res.

[46] Dresner-Pollak R, Gelb N, Rachmilewitz D, Karmeli F, Weinreb M (2004). Interleukin 10-deficient mice develop osteopenia, decreased bone formation, and mechanical fragility of long bones.. Gastroenterology.

[47] Evans KE, Fox SW (2007). Interleukin-10 inhibits osteoclastogenesis by reducing NFATc1 expression and preventing its translocation to the nucleus.. BMC Cell Biol.

[48] Chua KN, Chai C, Lee PC, Tang YN, Ramakrishna S (2006). Surface-aminated electrospun nanofibers enhance adhesion and expansion of human umbilical cord blood hematopoietic stem/progenitor cells.. Biomaterials.

[49] Sheng MH, Baylink DJ, Beamer WG, Donahue LR, Rosen CJ (1999). Histomorphometric studies show that bone formation and bone mineral apposition rates are greater in C3H/HeJ (high-density) than C57BL/6J (low-density) mice during growth.. Bone.

[50] McMichael BK, Cheney RE, Lee BS (2010). Myosin X regulates sealing zone patterning in osteoclasts through linkage of podosomes and microtubules.. J Biol Chem.

